# Visfatin is negatively associated with coronary artery lesions in subjects with impaired fasting glucose

**DOI:** 10.1515/med-2022-0540

**Published:** 2022-09-05

**Authors:** Fei Xu, Xiang Ning, Tong Zhao, Qinghua Lu, Huiqiang Chen

**Affiliations:** Department of Cardiology, The Second Hospital of Shandong University, Shandong University, Ji-nan, Shandong Province, China

**Keywords:** visfatin, prediabetes, impaired fasting glucose, coronary artery disease

## Abstract

It is not determined whether serum visfatin levels are related to the presence and severity of coronary artery disease (CAD) in non-diabetic subjects. In this study, a total of 65 consecutive non-diabetic participants who underwent coronary angiography were enrolled. Serum visfatin and fasting glucose, as well as the serum total cholesterol, low-density lipoprotein cholesterol, high-density lipoprotein cholesterol, and triglyceride, were measured in all participants before the procedure. The extent of coronary artery lesions was determined by Gensini score. Serum visfatin levels were significantly lower in patients with CAD compared to participants with normal coronary arteries. Inversely, the circulating levels of fasting glucose were found to be elevated in patients with CAD compared with the control subjects. Multivariable logistic regression analysis demonstrated that visfatin and impaired fasting glucose (IFG) were independently associated with the presence of CAD in non-diabetics. No significant relationship was found between serum visfatin and fasting glucose levels in IFG subjects. However, there was a negative association between visfatin concentrations and Gensini score in participants with IFG. Both circulating visfatin concentrations and IFG are independently associated with CAD in non-diabetics. Serum visfatin levels are negatively related to the angiographic severity of CAD in subjects with IFG.

## 1 Introduction

It is well established that diabetes mellitus is not only a risk equivalent of coronary artery disease (CAD) but also an independent risk factor for cardiovascular disease [1]. Prediabetes, which is defined by the presence of impaired fasting glucose (IFG) and/or impaired glucose tolerance and/or HbA1C levels ranging from 5.7 to 6.4% [2], is also an important risk factor for the development of cardiovascular disease [3]. In recent years, the impact of prediabetic state on coronary atherosclerosis is attracting more attention. High fasting blood glucose (FBG) is independently associated with the severity of coronary heart disease in prediabetic patients with glycosylated hemoglobin 5.7–6.4% [4]. Like diabetes, prediabetes is correlated with high atherosclerotic burden and the complexity of coronary artery lesions, which indicates that the prediabetic state might have the same effect as diabetes on coronary atherosclerosis [5,6]. Thus, due attention should be attached to the coronary atherosclerosis in subjects with prediabetes.

Visfatin, also known as pre-B-cell colony-enhancing factor or nicotinamide phosphoribosyltransferase, is an adipocytokine that is mainly produced in visceral fat tissue [7]. Visfatin might be involved in the development and progression of diabetes and atherosclerosis independently [8]. In fact, a growing number of evidence suggest that visfatin might take a part in the pathogenesis of atherosclerosis in diabetic patients. For example, high serum visfatin was not only significantly correlated with advanced carotid atherosclerosis [9] but also an independent predictor for the presence of peripheral atherosclerotic plaques in type 2 diabetic patients [10]. Further studies demonstrated that serum visfatin levels were higher in type 2 diabetic patients with CAD compared to those without CAD [11,12,13]. These findings signify that visfatin might be a biomarker or a promoting factor of the cardiovascular complications in patients with type 2 diabetes. However, the role of visfatin in cardiovascular complications of non-diabetics is not determined.

In the present study, we aimed to investigate the association of serum visfatin and the lesions of coronary artery in non-diabetics.

## 2 Patients and methods

### 2.1 Patient selection

The subjects were selected from inpatients with the symptoms of chest discomfort who underwent coronary angiography at the Second Hospital of Shandong University in China from November 2014 to December 2014. The present study did not include the patients with coronary artery spasm angina, valvular heart disease, systemic inflammatory disease, autoimmune disorder, neoplastic disease, severe hepatic, and renal dysfunction. Sixty-five consecutive non-diabetic patients were included in this study, in which the experimental group included 39 patients with acute coronary syndrome while the control group consisted of 26 subjects with angiographically normal coronary arteries. The study protocol was approved by the hospital ethics review board (The Second Hospital of Shandong University, Ji-nan, China) according to the Declaration of Helsinki. Informed written consent was obtained from each patient.

### 2.2 Blood sampling and definitions

All blood samples were collected after an overnight fast (12 h) during hospital stay for the measurement of FBG, visfatin, TC, low-density lipoprotein cholesterol (LDL-C), high-density lipoprotein cholesterol (HDL-C), and total triglyceride (TG). Visfatin was analyzed using a commercially available ELISA kit (Cloud-Clone Corp., Wuhan, Hubei, China). Dyslipidemia was defined as a fasting concentrations TC ≥ 6.22 mmol/L, LDL-C ≥ 4.14 mmol/L, HDL-C ≤ 1.04 mmol/L, or TG ≥ 2.26 mmol/L or having a history of dyslipidemia or the use of lipid-lowering medications [14]. Smoking status was confirmed by the medical history. Hypertension was defined as systolic blood pressure ≥140 mmHg and/or diastolic blood pressure ≥90 mmHg, or having a history of hypertension or current treatment with antihypertensive medications. The diagnostic criterion of CAD was the individuals with at least one obvious stenosis (>50%) of the lumen diameter in any of the major coronary arteries, including the left main coronary artery (LM), left anterior descending artery (LAD), left circumflex coronary artery (LCX), and right coronary artery (RCA), or main branches of the vascular system [15]. Body mass index (BMI) was calculated as weight in kilograms divided by the square of the height in meters (kg/m^2^).

### 2.3 Coronary angiography

Selective coronary angiographies were conducted with the standard Judkin’s technique by filming of multiple views of each blood vessel. Coronary angiograms were analyzed by two experienced interventional physicians who knew nothing about the clinical characteristics of the subjects.

The severity of coronary artery stenosis was evaluated by Gensini scoring system [15]. The Gensini score was computed by assigning a severity score to each coronary narrowing by both the degree of luminal stenosis and its geographic significance. The decrease in the luminal diameter of 25, 50, 75, 90, 99, and 100% occlusion was given the score of 1, 2, 4, 8, 16, and 32, respectively.

The score was then multiplied by a factor that symbolizes the functional importance of the lesion in the coronary arterial tree, for example, 5 for LM, 2.5 for the proximal LAD or LCX, 1.5 for the mid-LAD, and 1 for RCA or the distal LAD [15,16].

### 2.4 Statistical analysis

The normal distribution of the continuous variables was evaluated using the Shapiro–Wilk test. Continuous variables with a normal distribution were shown as mean ± SD and those with a non-Gaussian distribution were shown as median (25th–75th percentile). Student’s *t*-test was used for the comparison of normally distributed continuous numerical variables, and the Mann–Whitney *U* test was used for non-normally distributed numerical variables. The categorical variable was expressed as number of cases (*n*) and percentage (%), and categorical data between the groups were compared by chi-square test. Logistic regression analysis was carried out to determine the independent predictors of CAD. The covariates that associated with presence of CSF in the univariate model were included in the multivariate logistic regression analysis. Association of serum visfatin with fasting serum glucose and Gensini score were evaluated using bivariate Pearson’s correlation coefficients. Statistical analyses were performed using IBM SPSS (Statistical Package for the Social Sciences) for windows 23.0 statistical software package. A *P*-value of <0.05 was considered to be statistically significant.

## 3 Results

We studied a total of 65 consecutive non-diabetics. Forty-one (63%) of the patients were men. The average age and BMI of the patients were 61.2 ± 11.7 years and 23.1 ± 2.1 kg/m^2^, respectively. The percentage of patients with smoking, CAD, hypertension, and dyslipidemia was 38, 60, 51, and 26, respectively.

Table 1 shows the clinical and laboratory characteristics of the subjects classified according to CAD. As shown in the table, there were no significant differences in age, gender, BMI, smoking history or blood lipid between CAD patients and subjects with normal coronary arteries. The mean levels of fasting glucose in CAD subjects were significantly higher than participants with normal coronary arteries (*P* = 0.035). Likewise, the incidence of hypertension was more marked in CAD patients than in normal control (*P* = 0.044). However, the blood visfatin level was statistically lower in the CAD group than in the control group (*P* = 0.033).

**Table 1:** Baseline characteristics of study subjects

Variable	Non-CAD (*N* = 26)	CAD group (*N* = 39)	*P* value
Age (year)	57.50 ± 13.52	63.59 ± 9.82	0.055
Men (%)	14 (53.8)	27 (69.2)	0.294
BMI (kg/m^2^)	23.13 ± 1.93	23.14 ± 2.22	0.986
Smoking (%)	8 (30.8)	17 (43.6)	0.435
Hypertension (%)	9 (34.6)	24 (61.5)	**0.044**
SBP (mmHg)	133.92 ± 14.50	128.77 ± 17.92	0.226
DBP (mmHg)	78.50 (73.50–85.50)	75.00 (71.00–83.00)	0.348
Hyperlipidemia (%)	4 (15.4)	13 (33.3)	0.152
TC (mmol/L)	4.30 ± 0.81	4.33 ± 1.29	0.914
TG (mmol/L)	1.00 (0.83–1.49)	1.09 (0.86–1.48)	0.867
LDL-C (mmol/L)	2.20 ± 0.50	2.44 ± 0.82	0.145
HDL-C (mmol/L)	1.24 ± 0.20	1.13 ± 0.25	0.075
Fasting glucose (mmol/L)	5.17 ± 0.53	5.52 ± 0.77	**0.035**
Visfatin (ng/ml)	14.20 ± 11.01	9.34 ± 7.05	**0.033**

Continuous variables are shown as the mean ± SD or median (25th–75th percentiles). Categorical variable is expressed as number of cases (*n*) and percentage (%). Fonts in bold indicate statistical significance (*P* < 0.05).

IFG, impaired fasting glucose; BMI, body mass index; CAD, coronary artery disease; SBP, systolic blood pressure; DBP, diastolic blood pressure; TC, total cholesterol; TG, triglyceride; LDL-C, low-density lipoprotein cholesterol; HDL-C, high-density lipoprotein cholesterol.

To evaluate the possible confounding factors for the presence of CAD, logistic regression analysis was used. In the univariate analysis, age (*P* < 0.05), hypertension (*P* < 0.05), visfatin (*P* = 0.050), and IFG (*P* = 0.055) remained for the multivariate logistic regression analysis (Table 2). In the multivariate analysis, visfatin [odds ratio (OR) = 0.889, 95% CI 0.810–0.976; *P* = 0.013] and IFG (OR = 26.679, 95% CI 1.735–410.207; *P* = 0.019) were independently associated with the presence of CAD.

**Table 2:** Univariate and multivariate logistic regression analyses for independent predictors of the presence of CAD

	Univariate		Multivariate	
	OR (95% CI)	*P* value	OR (95% CI)	*P* value
Visfatin	0.938 (0.879–1.000)	0.050	0.889 (0.810–0.976)	**0.013**
IFG	3.833 (0.969–15.162)	0.055	26.679 (1.735–410.207)	**0.019**
Age	1.048 (1.001–1.097)	**0.045**	1.045 (0.993–1.101)	0.092
Hypertension	3.022 (1.075–8.499)	**0.036**	2.659 (0.795–8.888)	0.112
Male	1.929 (0.690–5.392)	0.211		
BMI	1.867 (0.568-6.129)	0.304		
Smoking	1.739 0.611–4.949)	0.300		
Dyslipidemia	2.750 (0.783–9.659)	0.115		

Fonts in bold indicate statistical significance (*P* < 0.05).

CAD, coronary artery disease; IFG, impaired fasting glucose; BMI, body mass index.

No significant association was found between serum visfatin levels and fasting glucose (*P* = 0.144) or Gensini score (*P* = 0.200) in CAD subjects. The correlation analysis for serum visfatin and fasting serum glucose or Gensini score in patients with IFG is included in Table 3. As tabulated in Table 3, circulating visfatin levels were negatively correlated with the Gensini scores in participants with IFG (*r* = −0.526, *P* = 0.037). However, no significant association was found between serum visfatin and fasting glucose levels in IFG subjects.

**Table 3:** Pearson correlation coefficients between visfatin and fasting glucose or gensini score in IFG subjects

Variables	Correlation coefficient	*P* value
Fasting glucose	–0.179	0.506
Gensini score	–0.526	**0.037**

Fonts in bold indicate statistical significance (*P* < 0.05).

IFG, impaired fasting glucose.

## 4 Discussion

The main finding of our study is that the serum visfatin levels are not only independently associated with CAD in non-diabetic patients but also negatively linked with the severity of coronary artery lesion in participants with IFG. It implies that visfatin might serve as an important protective biomarker and therapeutic target of coronary artery lesions in subjects with prediabetes in the future. Thus, our data support the idea that high visfatin levels are protective against coronary artery lesions in non-diabetics.

The notion above has been supported by previous studies. For example, the increased expression of visfatin in acute coronary syndrome patients might exert a protective effect by the upregulation of the NAMPT/NAD^+^/Sirt1 signaling pathway [17]. Visfatin could counteract H2O2-induced apoptotic damage in H9c2 cardiomyocytes via AMPK activation [18] while the pharmacological inhibition of Nampt could reduce neutrophilic inflammation- and oxidative stress-mediated tissue damage in early phases of reperfusion after a myocardial infarction [19]. However, there are studies demonstrating the important links between visfatin and inflammation, endothelial dysfunction, atherosclerosis, and plaque instability in CAD [20,21]. More studies are required to clarify the exact role and mechanisms of visfatin in the coronary atherosclerosis in IFG patients.

So far as we know, the role of visfatin in coronary atherosclerosis is not fully understood [20,21]. The study conducted by Darabi et al. found that serum visfatin was significantly higher in acute coronary syndrome patients than the stable CAD patients [22], indicating that visfatin might play an important part in the plaque instability and inflammation of coronary atherosclerosis. The circulating visfatin levels are significantly higher in acute ST-elevation myocardial infarction patients and the increased serum visfatin might be closely associated with the degree of myocardial damage [23]. Recently, the meta-analysis by Yu et al. showed that the increased serum visfatin concentrations might be a risk marker of coronary heart disease, in which 15 articles involving 1,053 CAD cases and 714 controls were included [24]. Recent studies demonstrated that serum visfatin concentration has obviously positive correlation with CAD severity evaluated by SYNTAX score, Gensini score or the number of narrowed coronary arteries, respectively [23,25,26,27]. However, the negative association between visfatin and CAD or its severity also been reported [28,29]. Further studies are warranted to explore the dynamic alterations and significance of visfatin in patients with CAD in different clinical contexts including the atherosclerotic process of non-diabetics.

Our previous work demonstrated that hyperglycemia in non-diabetics is closely related with the complexity of coronary artery and the fasting glucose is an independent predictor for severe CAD in people with prediabetes [30]. In this study, we further discovered that IFG, a prediabetic state, is also independently associated with CAD. This is in line with the findings of Yang et al. in which both FBG and glycosylated hemoglobin are independently correlated with the severity of CAD in prediabetic patients with glycosylated hemoglobin 5.7–6.4% [4]. In addition, Muhammed et al. demonstrated that the complexity of CAD was higher in the prediabetic than in normoglycemic subjects and comparable with diabetics [5]. Furthermore, the study by Açar et al. found that the people with prediabetes and diabetes showed a higher proportion of patients with three-vessel diseases and higher CAD severity than normoglycemic subjects [6]. The studies above indicate the promoting action of the prediabetic status including IFG to the development of coronary heart disease.

The roles of adipokines including visfatin in the cardiovascular complications of diabetic patients are increasingly attracting worldwide attention and study [31]. In recent years, a growing number of evidence shows that serum visfatin levels were higher in type 2 diabetic patients with CAD compared to non-CAD control [11,12,13]. Visfatin might also be associated with the atherosclerotic lesions of non-diabetic patients considering that prediabetes has the same effect on coronary artery lesions as diabetes [5]. However, the above results from diabetic subjects might be not applicable to non-diabetics. For example, the study by Saddi-Rosa demonstrated that circulating visfatin levels were not significantly different in non-diabetic participants with or without CAD [11]. Here, our study revealed that circulating visfatin levels are decreased significantly in non-diabetic participants with CAD compared to non-CAD. These might signify the different alterations in serum visfatin levels in CAD patients with or without diabetes. Larger studies are needed to investigate the expression and dynamic alterations of visfatin levels in CAD subjects from prediabetes to diabetes.

The visfatin expression and serum levels are influenced by fat area and distribution, inflammatory state, renal function, iron metabolism, hormones, and so on [32]. Besides the factors above, as reported in the literature, the blood concentration of visfatin could be enhanced by hyperglycemia while the hyperglycemia-induced visfatin increase could be inhibited by exogenous hyperinsulinemia [33]. Serum visfatin was found to have insulin-mimetic action and increase with progressive β-cell deterioration [34,35]. Thus, the increase in serum visfatin in diabetics or pre-diabetics might be indicative of a disease of higher severity. In this study, no significant association was found between visfatin levels and fasting glucose in CAD subjects with or without IFG. This might be related to the preserved endogenous insulin secretion and relatively short exposure to hyperglycemia in patients with prediabetes [36]. Recent clinical trial by Yang et al. demonstrated that purified anthocyanins’ supplementation for 12 weeks decreased serum visfatin in subjects with prediabetes or newly diagnosed diabetes [37]. Thus, elucidating the effect of anthocyanins supplementation on coronary atherosclerosis might help clarifying the relationship of visfatin and blood glucose in CAD progression. However, larger and prospective studies are needed in the future.

Previous research demonstrated that individual components of metabolic syndrome and their various combinations may have different contributions to CAD [38]. In our study, there are no significant differences in BMI, TC, TG, LDL-C or HDL-C between CAD and non-CAD subjects. Hypertension is an independent predictor for CAD in the univariate logistic regression analysis, but not in the multivariate logistic regression analysis.

Our study has several limitations. First, this study was a cross-sectional study and lacks long-term follow-up data. Second, there are a limited number of subjects in our study owing to the limited funding, which might reduce its statistical power. Third, all our subjects were undergoing coronary angiography, which might cause selection bias. Finally, there is a measurement bias since ELISA might be more sensitive but with a narrow detection range compared to enzyme immunoassay and radioimmunoassay [39].

To sum up, serum visfatin levels and IFG were independent predictive factors of the presence of CAD in non-diabetics. Visfatin levels were negatively associated with CAD severity in IFG subjects; thus, visfatin might play a protective part in the development of coronary atherosclerosis in participants with prediabetes.

## References

[1] Jung CH, Mok JO. Recent updates on vascular complications in patients with Type 2 diabetes mellitus. Endocrinol Metab. 2020;35(2):260–71. 10.3803/EnM.2020.35.2.260 PMC7386121 32615710

[2] Amer Diabet A. Classification and diagnosis of diabetes: Standards of medical care in diabetes-2018. Diabetes Care. 2018;41:S13–27. 10.2337/dc18-S002 29222373

[3] Zand A, Ibrahim K, Patham B. Prediabetes: Why should we care? Methodist DeBakey Cardiovasc J. 2018;14(4):289–97. 10.14797/mdcj-14-4-289 PMC6369626 30788015

[4] Yang J, Zhou Y, Zhang T, Lin X, Ma X, Wang Z, et al. Fasting blood glucose and HbA1c correlate with severity of coronary artery disease in elective PCI patients with HbA1c 5.7% to 6.4. Angiology. 2020;71(2):167–74. 10.1177/0003319719887655 31749367

[5] Muhammed A, Zaki MT, Elserafy AS, Amin SA. Correlation between prediabetes and coronary artery disease severity in patients undergoing elective coronary angiography. Egypt heart J. 2019;71(1):34. 10.1186/s43044-019-0034-y PMC6934639 31883041

[6] Açar B, Ozeke O, Karakurt M, Ozen Y, Ozbay MB, Unal S, et al. Association of prediabetes with higher coronary atherosclerotic burden among patients with first diagnosed acute coronary syndrome. Angiology. 2019;70(2):174–80. 10.1177/0003319718772420 29695169

[7] Dakroub A, Nasser SA, Younis N, Bhagani H, Al-Dhaheri Y, Pintus G, et al. Visfatin: A possible role in cardiovasculo-metabolic disorders. Cells. 2020;9(11):2444. 10.3390/cells9112444 PMC7696687 33182523

[8] Chang YH, Chang DM, Lin KC, Shin SJ, Lee YJ. Visfatin in overweight/obesity, type 2 diabetes mellitus, insulin resistance, metabolic syndrome and cardiovascular diseases: A meta-analysis and systemic review. Diabetes/metabolism Res Rev. 2011;27(6):515–27. 10.1002/dmrr.1201 21484978

[9] Kadoglou NP, Sailer N, Moumtzouoglou A, Kapelouzou A, Tsanikidis H, Vitta I, et al. Visfatin (nampt) and ghrelin as novel markers of carotid atherosclerosis in patients with type 2 diabetes. Exp Clin Endocrinol Diabetes. 2010;118(2):75–80. 10.1055/s-0029-1237360 19834878

[10] Zheng LY, Xu X, Wan RH, Xia S, Lu J, Huang Q. Association between serum visfatin levels and atherosclerotic plaque in patients with type 2 diabetes. Diabetol Metab Syndrome. 2019;11:60. 10.1186/s13098-019-0455-5 PMC6657107 31367237

[11] Saddi-Rosa P, Oliveira C, Crispim F, Giuffrida FM, de Lima V, Vieira J, et al. Association of circulating levels of nicotinamide phosphoribosyltransferase (NAMPT/Visfatin) and of a frequent polymorphism in the promoter of the NAMPT gene with coronary artery disease in diabetic and non-diabetic subjects. Cardiovasc Diabetol. 2013;12:119. 10.1186/1475-2840-12-119 PMC3765274 23968400

[12] Chen L, Wei B, Xu L, Wu Y. The association of inflammatory markers and periodontal indexes with the risk of coronary heart disease in Chinese patients with type 2 diabetes mellitus. Diabetes Res Clin Pract. 2018;135:37–44. 10.1016/j.diabres.2017.10.008 29111278

[13] Ahmed HH, Shousha WG, El-Mezayen HA, Emara IA, Hassan ME. New Biomarkers as prognostic factors for cardiovascular complications in type 2 diabetic patients. Indian J Clin Biochem. 2020;35(1):54–62. 10.1007/s12291-018-0784-4 PMC6995459 32071496

[14] Zhu JR, Gao RL, Zhao SP, Lu GP, Zhao D, Li JJ. Chinese guidelines for the management of dyslipidemia in adults Joint committee for guideline revision. J Geriatric Cardiol. 2016;15(1):1–29. 10.11909/j.issn.1671-5411.2018.01.011 PMC5803534 29434622

[15] Chen J, Chen MH, Li S, Guo YL, Zhu CG, Xu RX, et al. Usefulness of the neutrophil-to-lymphocyte ratio in predicting the severity of coronary artery disease: A Gensini score assessment. J Atheroscler Thromb. 2014;21(12):1271–82. 10.5551/jat.25940 25069816

[16] Gensini GG. A more meaningful scoring system for determining the severity of coronary heart disease. Am J Cardiol. 1983;51(3):606. 10.1016/s0002-9149(83)80105-2 6823874

[17] Zhang CX, Zhu R, Wang HP, Tao QS, Lin XH, Ge SL, et al. Nicotinamide phosphate transferase (NAMPT) increases in plasma in patients with acute coronary syndromes, and promotes macrophages to M2 polarization. Int Heart J. 2018;59(5):1116–22. 10.1536/ihj.17-363 30158377

[18] Xiao J, Sun B, Li M, Wu Y, Sun XB. A novel adipocytokine visfatin protects against H2O2-induced myocardial apoptosis: A missing link between obesity and cardiovascular disease. J Cell Physiol. 2013;228(3):495–501. 10.1002/jcp.24257 23065734

[19] Montecucco F, Bauer I, Braunersreuther V, Bruzzone S, Akhmedov A, Luscher TF, et al. Inhibition of nicotinamide phosphoribosyltransferase reduces neutrophil-mediated injury in myocardial infarction. Antioxid Redox Signal. 2013;18(6):630–41. 10.1089/ars.2011.4487 PMC3549207 22452634

[20] Dakroub A, Nasser SA, Kobeissy F, Yassine HM, Orekhov A, Sharifi-Rad J, et al. Visfatin: An emerging adipocytokine bridging the gap in the evolution of cardiovascular diseases. J Cell Physiol. 2021;236(9):6282–96. 10.1002/jcp.30345 33634486

[21] Hognogi LDM, Simiti LV. The cardiovascular impact of visfatin – an inflammation predictor biomarker in metabolic syndrome. Clujul Med. 19572016;89(3):322–6. 10.15386/cjmed-591 PMC4990425 27547049

[22] Darabi F, Aghaei M, Movahedian A, Elahifar A, Pourmoghadas A, Sarrafzadegan N. Association of serum microRNA-21 levels with Visfatin, inflammation, and acute coronary syndromes. Heart Vessel. 2017;32(5):549–57. 10.1007/s00380-016-0913-z 27785570

[23] Lu LF, Wang CP, Yu TH, Hung WC, Chiu CA, Chung FM, et al. Interpretation of elevated plasma visfatin concentrations in patients with ST-elevation myocardial infarction. Cytokine. 2012;57(1):74–80. 10.1016/j.cyto.2011.10.015 22137121

[24] Yu F, Li J, Huang Q, Cai H. Increased peripheral blood visfatin concentrations may be a risk marker of coronary artery disease: A meta-analysis of observational studies. Angiology. 2018;69(9):825–34. 10.1177/0003319718771125 29706084

[25] Duman H, Ozyildiz AG, Bahceci I, Duman H, Uslu A, Ergul E. Serum visfatin level is associated with complexity of coronary artery disease in patients with stable angina pectoris. Ther Adv Cardiovasc Dis. 2019;13:1753944719880448. 10.1177/1753944719880448 PMC6778987 31588856

[26] Guan J, Wu L, Xiao Q, Pan L. Levels and clinical significance of serum homocysteine (Hcy), high-density lipoprotein cholesterol (HDL-C), vaspin, and visfatin in elderly patients with different types of coronary heart disease. Ann Palliat Med. 2021;10(5):5679–86. 10.21037/apm-21-1001 34107717

[27] Fadaei R, Parvaz E, Emamgholipour S, Moradi N, Vatannejad A, Najafi M, et al. The mRNA expression and circulating levels of visfatin and their correlation with coronary artery disease severity and 25-Hydroxyvitamin D. Hormone Metab Res. 2016;48(4):269–74. 10.1055/s-0035-1564133 26466019

[28] Choi KM, Lee JS, Kim EJ, Baik SH, Seo HS, Choi DS, et al. Implication of lipocalin-2 and visfatin levels in patients with coronary heart disease. Eur J Endocrinol. 2008;158(2):203–7. 10.1530/EJE-07-0633 18230827

[29] Kadoglou NPE, Gkontopoulos A, Kapelouzou A, Fotiadis G, Theofilogiannakos EK, Kottas G, et al. Serum levels of vaspin and visfatin in patients with coronary artery disease-Kozani study. Clinica Chim Acta. 2011;412(1–2):48–52. 10.1016/j.cca.2010.09.012 20850423

[30] Zhao T, Gong HP, Dong ZQ, Du YM, Lu QH, Chen HQ. Predictive value of fasting blood glucose for serious coronary atherosclerosis in non-diabetic patients. J Int Med Res. 2019;47(1):152–8. 10.1177/0300060518798252 PMC6384470 30208754

[31] Jaganathan R, Ravindran R, Dhanasekaran S. Emerging role of adipocytokines in type 2 diabetes as mediators of insulin resistance and cardiovascular disease. Can J Diabetes. 2018;42(4):446–56. 10.1016/j.jcjd.2017.10.040 29229313

[32] Filippatos TD, Randeva HS, Derdemezis CS, Elisaf MS, Mikhailidis DP. Visfatin/PBEF and atherosclerosis-related diseases. Curr Vasc Pharmacol. 2010;8(1):12–28. 10.2174/157016110790226679 19485930

[33] Haider DG, Schaller G, Kapiotis S, Maier C, Luger A, Wolzt M. The release of the adipocytokine visfatin is regulated by glucose and insulin. Diabetologia. 2006;49(8):1909–14. 10.1007/s00125-006-0303-7 16736128

[34] Xie H, Tang SY, Luo XH, Huang J, Cui RR, Yuan LQ, et al. Insulin-like effects of visfatin on human osteoblasts. Calcif Tissue Int. 2007;80(3):201–10. 10.1007/s00223-006-0155-7 17340225

[35] Lopez-Bermejo A, Chico-Julia B, Fernandez-Balsells M, Recasens M, Esteve E, Casamitjana R, et al. Serum visfatin increases with progressive beta-cell deterioration. Diabetes. 2006;55(10):2871–5. 10.2337/db06-0259 17003355

[36] Kaminska A, Kopczynska E, Bielinski M, Borkowska A, Junik R. Visfatin concentrations in obese patients in relation to the presence of newly diagnosed glucose metabolism disorders. Endokrynol Polska. 2015;66(2):108–13. 10.5603/EP.2015.0016 25931039

[37] Yang L, Qiu Y, Ling W, Liu Z, Yang L, Wang C, et al. Anthocyanins regulate serum adipsin and visfatin in patients with prediabetes or newly diagnosed diabetes: A randomized controlled trial. Eur J Nutr. 2021;60(4):1935–44. 10.1007/s00394-020-02379-x 32930848

[38] Zhang YF, Hong J, Gu WQ, Gui MH, Chen Y, Zhang Y, et al. Impact of the metabolic syndrome and its individual components on risk and severity of coronary heart disease. Endocrine. 2009;36(2):233–8. 10.1007/s12020-009-9214-y 19618299

[39] Korner A, Garten A, Bluher M, Tauscher R, Kratzsch J, Kiess W. Molecular characteristics of serum visfatin and differential detection by immunoassays. J Clin Endocrinol Metab. 2007;92(12):4783–91. 10.1210/jc.2007-1304 17878256

